# Editorial: Introducing novel trends in the nutrition of monogastric farm animals for the production of high-quality livestock products

**DOI:** 10.3389/fvets.2024.1514197

**Published:** 2025-01-07

**Authors:** Vassilios Dotas, George Symeon, Karoly Dublecz

**Affiliations:** ^1^School of Agriculture, Aristotle University of Thessaloniki, Thessaloniki, Greece; ^2^Research Institute of Animal Science, Hellenic Agricultural Organization - ELGO, Athens, Greece; ^3^Institute of Physiology and Nutrition, Hungarian University of Agricultural and Life Sciences, Gödöllo, Hungary

**Keywords:** monogastric animals, pigs, poultry, nutrition, performance, meat and egg quality, animal welfare, environment

The rearing of monogastric farm animals, especially pigs and poultry, is a significant animal husbandry activity worldwide, contributing ~75% to global meat production, fully meeting the demand for eggs, and providing animal protein sources of high nutritional and biological value (1). Pig and poultry production has shown adaptability to changing conditions and consumer concerns, through increased productivity, diverse products, and alternative production systems. However, challenges remain, particularly regarding climate change, welfare concerns, and production sustainability, especially given the ongoing energy and economic crises and food security threats. This Research Topic presents 10 papers covering novel aspects of these issues.

One of the key challenges in farm animal production in Europe is climate change adaptation. It affects animals through severe heat stress and feedstuff production. Corn, crucial in many countries, faces severe losses from heat and drought. Using alternative, stress-resistant cereal grains may be a solution. Barley, rye, and oats are less sensitive but contain fibers that can have depressive effects beyond certain inclusion rates. Lingens et al. investigated broken rye as a substitute for whole wheat with turkeys, finding that rye up to 10% can replace wheat and increase production sustainability. This aligns with earlier findings (2, 3), showing that feeding whole or broken cereal grains saves grinding costs, stimulates gizzard function, and positively affects digestion.

Another important issue in Europe is increasing protein self-sufficiency in animal nutrition. Importing soybean meal creates insecurity and a high carbon footprint. Other feedstuffs are available (legume seeds, extracted meals, dried distilled grains with solubles - DDGS, etc.), but their maximum inclusion rates are not always clear. Such et al. investigated the use of extracted sunflower meal (SFM) to replace soybean in the diets of pullets and laying hens. Results showed that SFM, even at a 30% inclusion rate, did not reduce ileal amino acid absorption and could fully substitute soybean.

A paper also explored low-protein diets for broiler chickens. While many results are available, practical implementation is constrained by special requirements and ratios of some essential and non-essential amino acids, such as threonine or glycine (4). Since crystalline glycine is not allowed in Europe, feedstuffs with higher glycine content can improve the efficiency of low-protein diets. Strifler et al. showed that increasing the threonine-to-lysine ratio or using meat meal as a glycine source improved weight gain and feed conversion of broiler chickens in the grower and finisher phases. However, changes in the starch-to-protein ratio in low-protein diets increased abdominal fat in birds.

Research has also focused on balancing growth performance and intestinal health in weaned piglets. Correia et al. examined different crude protein (CP) levels and supplementation with non-essential amino acids (NEAAs) like arginine, glutamine, and glutamate. Lowering CP in diets is often necessary to reduce nitrogen excretion but may compromise growth. The study found that while higher CP diet (24%) improved feed conversion, lower CP diet (18%) with NEAAs supported intestinal health by increasing villus height and goblet cells, and reducing inflammatory markers. This suggests that NEAA supplementation can mitigate the negative effects of low CP diets on gut health.

Wang et al. explored alternative feed ingredients, using storage japonica brown rice (SJBR) as a corn substitute in growing-finishing Min pigs. The aim was to improve feed efficiency while reducing costs. Bile acids were added to enhance fat digestion. Results indicated SJBR improved feed conversion ratios and increased beneficial gut bacteria. Adding bile acids further reduced back fat thickness and improved lipid metabolism, demonstrating the potential of these strategies for improving production efficiency while maintaining meat quality.

Yin et al. focused on enzyme supplementation to improve nutrient utilization and bone health in pigs. They evaluated phytase and a multi-carbohydrase-phytase complex (MCPC) on phosphorus digestibility and bone mineral content in growing pigs fed corn- or wheat-based diets. Enzyme supplementation, particularly MCPC, enhanced phosphorus absorption and bone strength while positively affecting gut microbial diversity. These findings emphasize the role of enzymes in boosting growth performance and nutrient utilization.

In livestock farming, nutrition is often the deciding factor for operational efficiency, both in terms of cost and productivity. A considerable amount of research is directed toward using alternative feedstuffs and feed additives to promote animal production in the face of modern challenges like climate change and rising raw material prices. Izuddin et al. evaluated the effects of different oil sources (crude palm oil, red palm oil, refined palm oil, palm kernel oil, or soybean oil) on blood lipid profiles, fatty acid deposition, and hepatic lipid and lipoprotein gene expression in laying hens. Their findings showed that all oils were suitable for use, but soybean oil increased omega-3 and omega-6 fatty acids in tissues. The choice of oil should reflect the producer's target, such as enhancing specific fatty acids in eggs and meat or reducing costs by using cheaper oils like crude palm oil.

Regarding feed additives, Almeldin et al. and Suliman et al. worked with broilers, using iron nanoparticles and algae, as well as nano-emulsified plant oil and probiotics, to promote meat production and quality. Almeldin et al. showed that green Nano-Fe up to 40 mg/kg, using 1 g/kg *Halimeda opuntia* as a carrier, or alone, enhanced broiler performance, carcass traits, and meat quality. Suliman et al. found that supplementing male broilers with essential oils and probiotics improved meat chewiness by reducing cohesiveness and hardness, while increasing springiness. Moniruzzaman et al. examined the effects of curcumin nanospheres in finishing pigs, finding promising results for enhanced growth, immunity, and gastrointestinal health.

In conclusion, the papers presented in this Research Topic offer valuable insights into innovative approaches in monogastric nutrition. The research emphasizes optimizing feed ingredients, alternative proteins, and dietary supplements to address climate change, economic pressures, and shifting consumer demands. By improving growth performance, nutrient utilization, and animal health, sustainable livestock farming becomes attainable. Despite the progress made, ongoing research is vital to further refine these strategies, ensuring animal production evolves in line with global sustainability goals. Together, these studies highlight the crucial role of nutrition in shaping the future of animal production.
